# Experimental Investigation on Seismic Performance of Non-Uniformly Corroded RC Moment-Resisting Frames

**DOI:** 10.3390/ma16072649

**Published:** 2023-03-27

**Authors:** Shang Chen, Huanjun Jiang

**Affiliations:** 1 State Key Laboratory for Disaster Reduction in Civil Engineering, Shanghai 200092, China; 2 Department of Disaster Mitigation for Structures, College of Civil Engineering, Tongji University, Shanghai 200092, China

**Keywords:** RC structure, electrochemical accelerated corrosion, non-uniform corrosion, corrosion of longitudinal steel reinforcement, seismic performance

## Abstract

To investigate the effect of non-uniform corrosion on the seismic performance of reinforced concrete (RC) frame structures, seven RC frame specimens, including one uncorroded and six corroded frames, were tested under quasi-static loading. The damage modes, force–displacement hysteresis curves and skeleton curves, stiffness degradation, ductility and the energy dissipation capacity of specimens were studied. The influences of the corrosion degree, non-uniform corrosion characteristic value, and axial compression ratio on the seismic performance of the specimens were analyzed. The test results show that non-uniform corrosion of longitudinal bars has a significant effect on the development of seismic damage in RC frames. In comparison with non-corroded RC frames, the loading carrying capacity of corroded frames with non-uniform corrosion characteristics values of 0.18, 0.59, and 0.72 decreased by 8.5%, 14.8%, and 22.3%, the displacement ductility ratios decreased by 6.7%, 8.7%, and 10.0%, and the total cumulative energy dissipation at ultimate displacement values decreased by 24%, 41%, and 54%. For corroded frames with low axial compressive ratios, the loading carrying capacity and energy dissipation capacity rose with the increase in the axial compression ratio.

## 1. Introduction

Reinforcement corrosion is one of the major causes of the loss of loading capabilities, decreasing the service life of RC structures. Corrosion of reinforcement will not only lead to a reduction on the cross-section [1] and the degradation of mechanical properties of reinforcement [2], but it will also lead to degradation of the bond between reinforcement and concrete [3]. There is a large number of existing buildings that have been built in the last century in earthquake-prone areas [4,5], which are exposed to both corrosion effects and the risk of seismic hazards. It will eventually lead to deterioration of the properties of RC structures [6], such as transformation of the damage mode [7,8], reduction of the bearing capacity [9,10] and a decline in ductility [11].

In 1988, Okada et al. [12] conducted low-cyclic repeated load tests on RC beams with longitudinal corroded cracks and found that the bearing capacity of corroded RC beams was reduced. Since then, many investigations have been carried out on the performance of corroded RC components. Tomes-Acosta et al. conducted tests on corroded beams under cyclic loading and found that the flexure stiffness of the corroded beam significantly decreased [13]. Meda et al. carried out cyclic loading tests on full-scale corroded column specimens and found that the bearing capacity and deformation capacity of the corroded column decreased [14]. Goksu et al. carried out quasi-static loading tests on six corroded columns and proposed a method to calculate the deformation capacity of corroded RC columns [15]. It was found by previous studies that steel reinforcement corrosion has significant influence on the seismic performance of RC members.

The reinforcement corrosion caused by chloride is always a stochastic process due to the heterogeneity of environment and the dispersion of concrete composition and the concrete cover thickness. The corrosion of reinforcement is always non-uniformly distributed in the structural members [16,17]. However, most of the previous studies assume that the steel reinforcement uniformly corrodes, which cannot precisely reflect the effect of corrosion on the seismic performance of the RC structure. A few researchers investigated the effect of non-uniform corrosion on the seismic performance of RC members, mainly focusing on three non-uniform types, i.e., the non-uniform corrosion of rebar cross-section [18,19], non-uniform corrosion along the axial direction of longitudinal reinforcement [20,21], and the non-uniform corrosion of rebars in different locations of the structural member [22,23,24]. The previous experimental studies focused on RC beams and columns. Ye et al. [22] conducted an experimental study on the shear behavior of corroded RC beams with different corrosion ratios for the stirrup and longitudinal reinforcement. The results showed that the corrosion of reinforcements in different locations might change the damage mode of RC beams. Yuan et al. [23] conducted tests on the seismic performance of six columns with non-uniform corrosion along the axial direction of longitudinal reinforcement. Li et al. [24] conducted cyclic loading tests on six columns with non-uniform corrosion of the longitudinal reinforcement at each side. Based on the test results, a bilateral failure criterion was established, considering the adverse effects of non-uniform corrosion. Previous studies mainly focused on corroded beams and columns at a component level, while there is only a limited amount of investigative information at the structural level. The non-uniform corrosion of reinforcement is common in RC structures. However, experimental studies on the seismic performance of non-uniformly corroded RC frames are presently limited.

The aim of this study is to investigate the effect of non-uniform corrosion on the seismic performance of RC moment-resisting frames. Seven RC frames, including six corroded frames with non-uniform corrosion of the longitudinal reinforcement on each side of the RC members and one uncorroded frame, were tested under cyclic lateral loading. The main variables studied were the average corrosion ratio of longitudinal reinforcement, the non-uniform corrosion characteristic value, and the axial compression ratio. The damage development, strength, force–displacement hysteresis behavior, deformation capacity, and energy dissipation capacity were studied.

## 2. Experimental Program

### 2.1. Specimen Information

**Figure 1:**
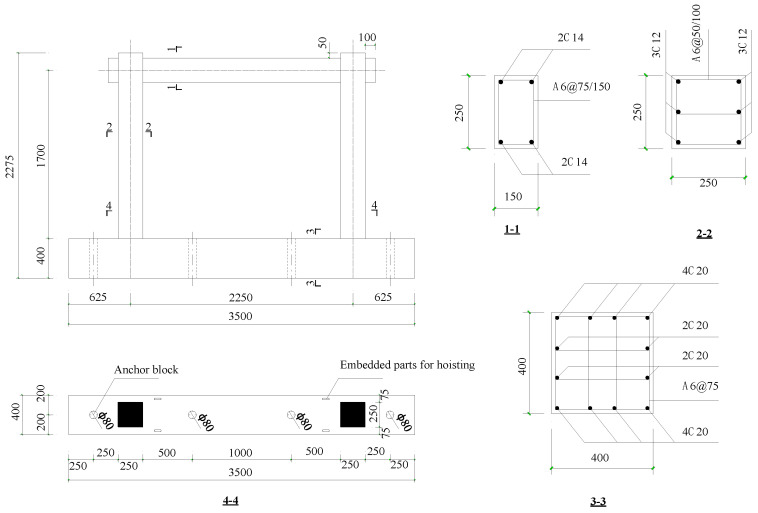
Dimensions and steel reinforcement of specimen (unit: mm).

The study of the seismic performance of reinforced concrete structures with non-uniform corrosion is important, not only for the evaluation of the seismic performance of existing structures, but also for the life-cycle design of new structures. In this study, a typical three-story RC moment frame structure was selected as the prototype structure and designed according to the current Chinese seismic design code (GB50011-2010) [25]. The prototype structure is sited in an earthquake-prone area with a seismic intensity of 7, a site soil class of III, and a design group of 1. The structural design was based on the principle of strong shear and weak flexure. The substructure of middle span of the first story of the frame structure was selected as the prototype structure for the scaled specimens. The dimension scale was 1:2. The dimensions and steel reinforcement details of the specimens are shown in Figure 1. The geometrical dimensions and reinforcement of all specimens were identical. The variable parameters included the average corrosion ratio of longitudinal reinforcement, the non-uniform corrosion characteristic value, and the axial compression ratio. The main parameters of the specimens are shown in Table 1. According to guidelines in the manual [26], a low corrosion level is defined as an average corrosion ratio of less than 5%, a medium corrosion level is a ratio between 5% and 10%, and a high corrosion ratio is defined as a ratio of more than 10%. Accordingly, in this study, four average corrosion ratios were considered, i.e., 0, 5%, 10%, and 15%. In total, seven specimens were constructed, including six non-uniformly corroded frames and one uncorroded frame.

RC specimens were constructed using the same batch of commercial concrete. The average cubic compressive strength after 28 days of standard curing was obtained: 38.8 MPa. The actual yield and ultimate strengths of the longitudinal reinforcement with a diameter of 12 mm were 487 MPa and 585 MPa, respectively. The actual yield and ultimate strength of the reinforcement with a diameter of 14 mm were 479 MPa and 680 MPa, respectively. The formula for calculating the non-uniform corrosion characteristic value is as follows:

(1) $$\rho =\frac{A_{corr,1}-A_{corr,2}}{A_{corr,all}}$$

where *A_corr_*_,1_ and *A_corr_*_,2_ are the larger and smaller corroded average loss area of reinforcement of two sides of the cross-section, respectively; *A_corr,all_* is the total average corroded loss area of reinforcement of the member.

### 2.2. Electrochemical Accelerated Corrosion

**Figure 2:**
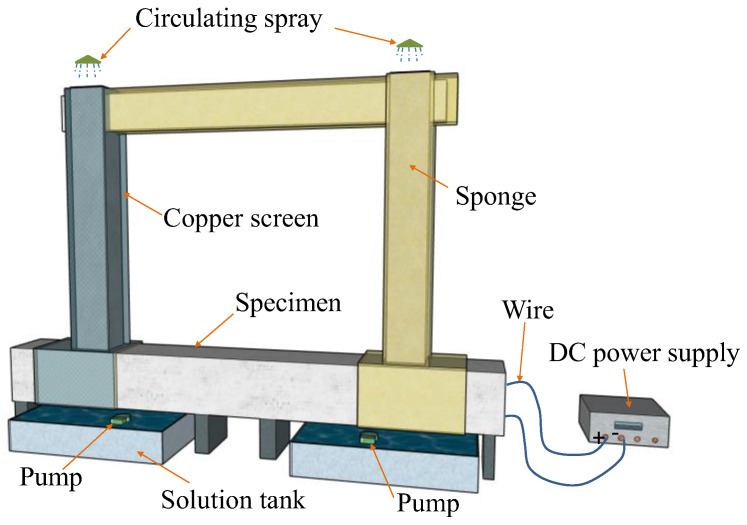
Schematic of accelerated corrosion system.

The accelerated corrosion method was used to accelerate the corrosion of reinforcement. The accelerated corrosion system is shown in Figure 2. Specimens were soaked in 5% NaCl solution in advance to make reinforcement easier to corrode when they were powered. Additionally, the specimens were subjected to accelerated electrochemical corrosion using a steady DC power device. The contact surfaces between stirrups and longitudinal reinforcements were insulated with plastic mats to avoid the interaction between them when an impressed current was applied. The mass of each reinforcement was weighed before accelerated corrosion. According to Faraday’s law and [27], the current design density of the target corroded longitudinal reinforcement in the specimen was 3 mA/cm^2^. Three DC power supplies were used to control the current in each specimen, and an independent DC power supply powered each member (Column A, Column B, and Beam). The longitudinal reinforcement in each member was connected in parallel. The target corrosion rate was controlled by controlling the energizing duration of each part.

### 2.3. Test Setup, Instrumentation, and Loading Protocol

**Figure 3:**
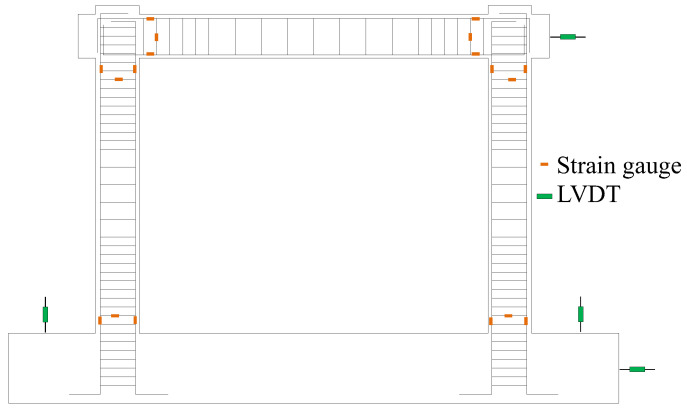
Layout of measuring points.

The arrangement of the measurement instruments is shown in Figure 3. These instruments were used to monitor the responses of specimens. Linear variable displacement transducers (LVDTs) in the horizontal direction were arranged at the top and base of the specimen to monitor horizontal loading displacement and possible sliding. LVDT for monitoring the displacement of the loading point was set at the height of 1700 mm from the base. Two vertical LVDTs of the base were used to monitor the possible rotation of the specimen base. Strain gauges were used to measure the strains of rebars in critical regions.

**Figure 4:**
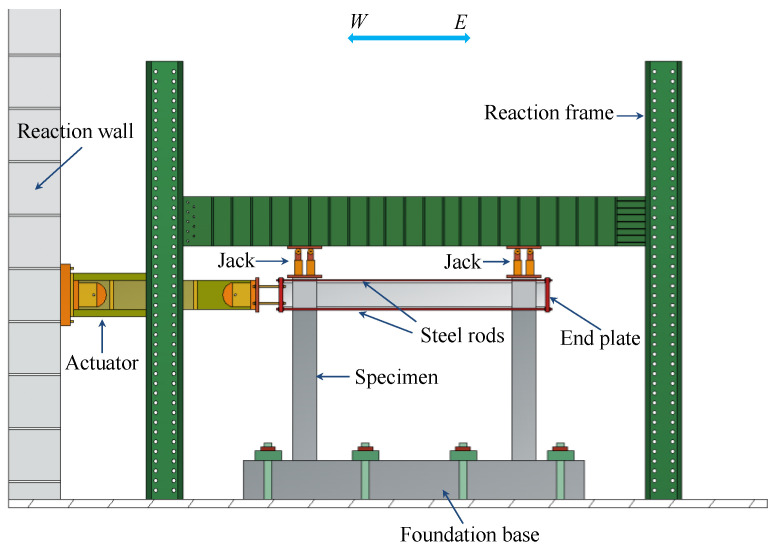
Test set-up.

**Figure 5:**
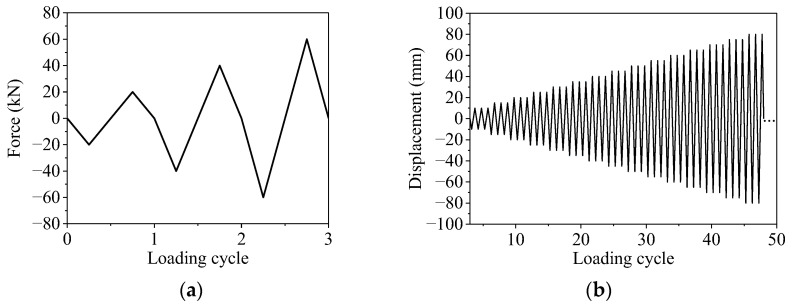
Lateral loading protocol: (**a**) force control stage; (**b**) displacement control stage.

The test was carried out on the specimens under cyclic loading. The test set-up is illustrated in Figure 4. The vertical load was applied at the top of two columns and kept constant using hydraulic jacks. The lateral load was applied the top of the specimen using an actuator. Strain gauges attached in advance to the reinforcement were used to monitor the yielding state of the specimen. The state when the longitudinal reinforcement reaches the yielding is defined as the yielding state of the specimen. Before the specimen yielded, the lateral load was applied in the force control mode, and one loading cycle was applied for each load amplitude. After the specimen yielded, it was loaded in the displacement control mode, and three loading cycles were applied for each displacement amplitude. The loading scheme is shown in Figure 5. Displacement, load, and strain were recorded during the entire experimental process using a data acquisition device.

## 3. Experimental Results

### 3.1. Damage Due to Corrosion

**Figure 6:**
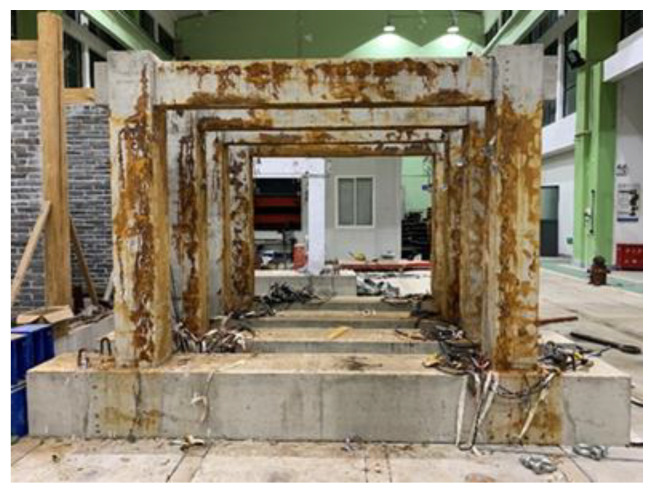
Appearance of corroded specimen.

**Figure 7:**
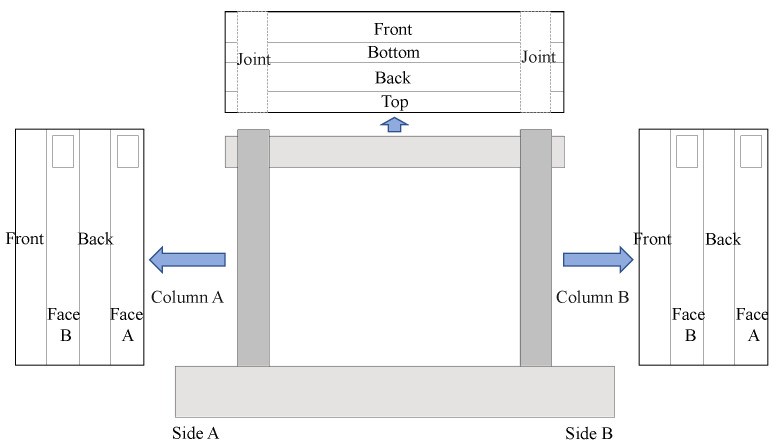
Expanded surface of specimen.

**Figure 8:**
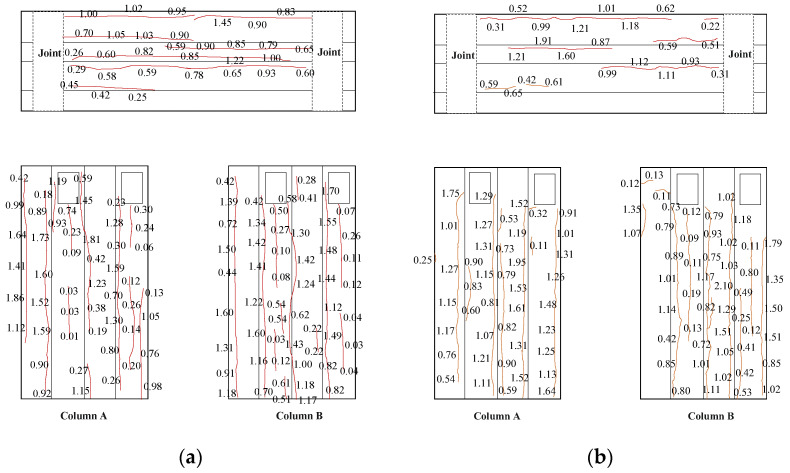
Distribution of corrosion expansion cracks of specimens. (unit: mm): (**a**) specimen S3; (**b**) specimen S7.

The damage due to corrosion in the specimens was evaluated after the completion of accelerated electrochemical corrosion. The form of corrosion damage is similar in all the corroded specimens, with corrosion expansion cracks having appeared along the longitudinal reinforcement at the corners of members. Corrosion products spilt out through the corrosion expansion cracks and dispersed into the solution tank with the circulating solution, as shown in Figure 6. The surface planar expansion diagram of the specimens is shown in Figure 7. The distribution of corrosion expansion cracks of two typical specimens before the loading is shown in Figure 8. The numbers in the diagram are the widths of corrosion expansion cracks.

In addition, to obtain the actual corrosion ratio of each specimen, after the loading test, the rebars were taken out from each specimen and partitioned into segments of equal length, washed with the acid, neutralized by lime water, and dried in a drying oven. The actual corrosion ratios of specimens are shown in Table 1.

### 3.2. Damage Evolution

**Figure 9:**
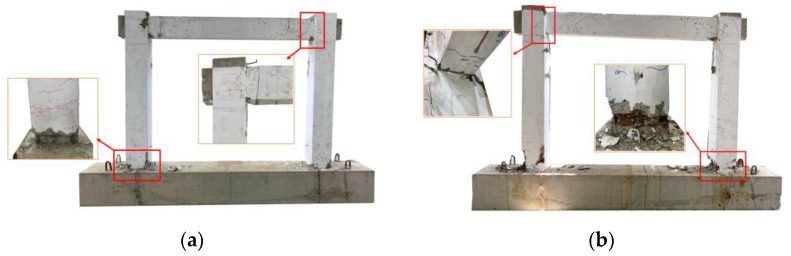
Failure modes of specimens: (**a**) specimen S1; (**b**) specimen S2.

All the specimens show flexural failure. The failure modes of two typical specimens in the ultimate limit state are shown in Figure 9. The state when the lateral force of the specimen in both directions was reduced to 85% of the peak load, it is defined as the ultimate limit state. Plastic hinges were formed at beam’s ends and column’s bottoms.

For the non-corroded specimen, S1, vertical cracks appeared first at the beam’s ends during the force control phase, and then horizontal cracks appeared at the column’s bottoms with the increase in the force. As the displacement amplitude increased to 15 mm, the longitudinal reinforcement at the beam’s ends and the bottom of column B yielded successively in the same cycle. When the displacement amplitude increased to 25 mm, the cover concrete at the beam’s end was crushed. When the displacement amplitude increased to 30 mm, the cover concrete at the column’s bottom was crushed. When the displacement amplitude increased to 50 mm, the cover concrete of the column’s bottom began to spall. When the displacement amplitude increased to 60 mm, the cover concrete of the beam’s end started to spall. When the displacement amplitude increased to 75 mm, the longitudinal reinforcement at the column’s bottom fractured. Until the end of the loading, there was no fracture of the steel reinforcement in the beam and no yielding of the stirrup in the beam and columns.

The damage development processes are similar for all corroded specimens, so only the damage evolution of the corroded specimen, S2, is introduced here as an example. During the force control stage, horizontal cracks appeared first at the columns’ bottoms, and then vertical flexural cracks occurred at the beam’s ends. When the loading displacement amplitude increased to 15 mm, the longitudinal reinforcement at the column’s bottoms yielded. When the loading displacement amplitude increased to 20 mm, crushing of the concrete cover at the column’s bottom was observed. When the loading displacement amplitude increased to 25 mm, crushing of the concrete cover at the beam’s end was observed. When the loading displacement amplitude increased to 45 mm, the cover concrete at the beam’s end spalled. When the loading displacement amplitude increased to 50 mm, the concrete of the beam cover spalled. The longitudinal reinforcement at the column’s bottom fractured when the loading displacement increased to 65 mm. Until the end of the loading experiment, the steel reinforcement in the beam remained unyielding and unfractured, and no yielding of the stirrup occurred.

The longitudinal reinforcement at beam’s ends and column’s bottoms of the uncorroded frame all yielded before failure. The longitudinal reinforcement at beam’s ends of all corroded frames, except specimen S3, did not yield during the test. The longitudinal reinforcement in the beam’s end in specimen S3 yielded under the compression at the loading displacement amplitude of 45 mm. The main reason for this phenomenon is that the corrosion leads to the degradation of the bond strength between the longitudinal reinforcement and the concrete, which makes the strain of the reinforcement develop more slowly, so that the strength of the reinforcement cannot be fully developed. Specimen S3 is the specimen with the smallest non-uniform corrosion characteristic value. The larger the non-uniform corrosion characteristic value is, the higher is the corrosion ratio is on one side of the RC member, which makes bond failure occur more easily.

In terms of damage distribution, the cracks in the uncorroded frames were more fully developed with a broader distribution. The cracks in the corroded frames were less developed. As the non-uniform corrosion characteristic value increased, the damage in the corroded specimens developed more quickly and was more concentrated. Compared to the uncorroded specimen, S1, as the non-uniform corrosion characteristic value increased, the displacement amplitudes for spalling of concrete at the column’s bottoms of specimens S3, S4, and S5 were 25%, 33%, and 42% smaller, respectively, and the displacement amplitudes for reinforcement fracture were 20%, 27%, and 33% smaller, respectively.

### 3.3. Hysteresis Behavior

**Figure 10:**
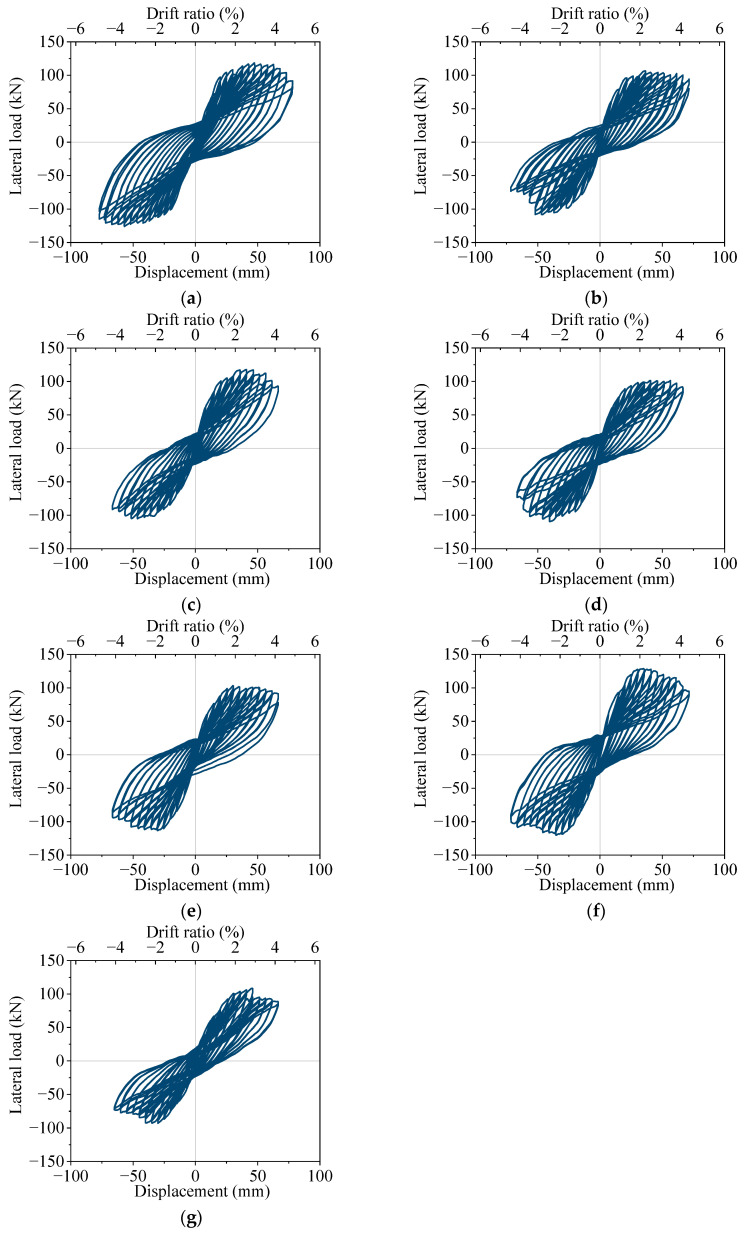
Lateral load–displacement hysteretic curves of specimens: (**a**) specimen S1; (**b**) specimen S2; (**c**) specimen S3; (**d**) specimen S4; (**e**) specimen S5; (**f**) specimen S6; (**g**) specimen S7.

Load–displacement hysteresis curves of the specimens are shown in Figure 10. For the uncorroded specimen, S1, after the lateral load reaches its peak value, the load decreases insignificantly with the increase in displacement amplitude until the longitudinal reinforcement fractured. For specimens S2, S4, and S7, the hysteresis loop area becomes significantly smaller with the increase in the average corrosion ratio. For specimens S3, S4, and S5, with the increase in the non-uniform corrosion characteristic value, the non-symmetry of the hysteresis curve becomes more significant, and the area of the hysteresis curve gradually becomes smaller.

**Figure 11:**
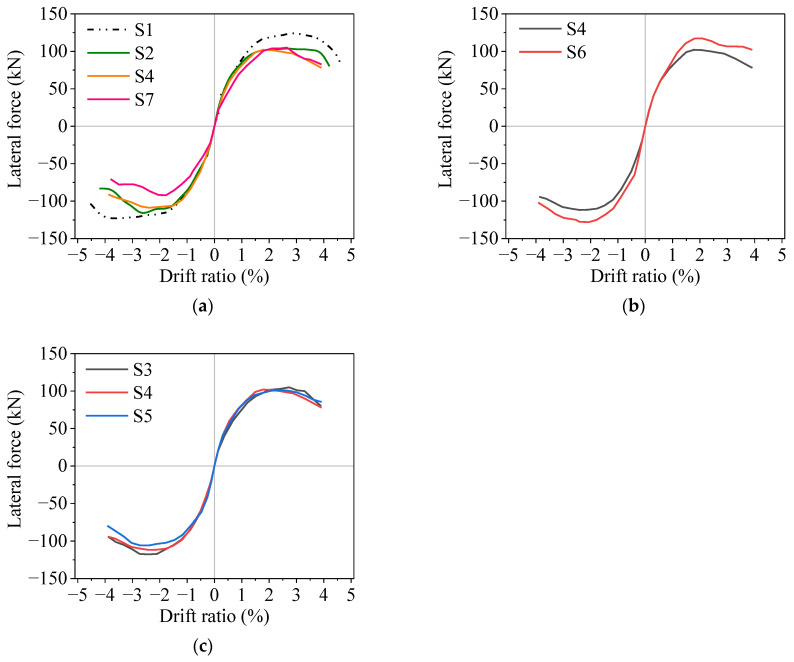
Lateral load–drift ratio skeleton curves of specimens: (**a**) different average corrosion ratio; (**b**) different axial compression ratio; (**c**) different non-uniform corrosion characteristic value.

Lateral force versus drift ratio skeleton curves of the specimens are shown in Figure 11. Table 2 shows the force and displacement at individual characteristic points and the ductility of the specimens. The yield displacement was calculated based on the equivalent elastic–plastic energy dissipation concept [28]. For specimens S2, S4, and S7, the maximum bearing capacity decreases significantly with the increase in the average corrosion ratio, and the bearing capacity in the negative loading direction is always lower than the that in the positive loading direction. For specimens S3, S4 and S5, with the increase in the non-uniform corrosion characteristics value, the bearing capacity decreases by 15.1%, 17.5%, and 21.6%, respectively, and the displacement ductility ratio decreases by 6.7%, 8.7%, and 10.0%, respectively, compared to those of the uncorroded specimen S1. When the lateral load reaches the maximum value, it decreases significantly with the increase in the loading displacement amplitude. For specimens S5 and S6, the specimen with larger axial force has a higher bearing capacity.

### 3.4. Stiffness Degradation

**Figure 12:**
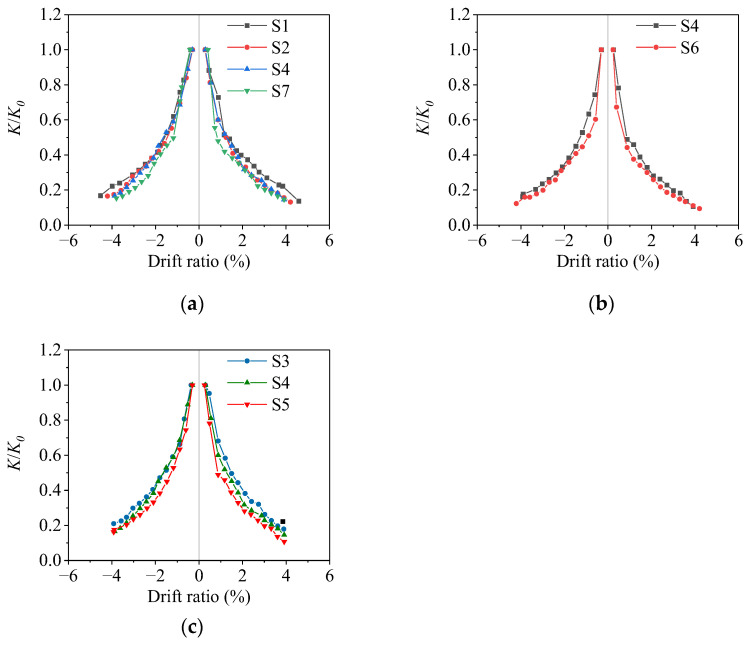
Stiffness degradation of specimens: (**a**) different average corrosion ratio; (**b**) different axial compression ratio; (**c**) different non-uniform corrosion characteristic value.

The degradation of effective stiffness of the specimen with the drift ratio is shown in Figure 12, where *K*_0_ is the initial stiffness and *K_i_* is the effective stiffness of the specimen at the loading displacement amplitude ∆*_i_*, which is calculated as

(2) $$K_{i}=\frac{F_{i}}{{∆}_{i}}$$

where *F_i_* is the peak load of the loading displacement amplitude ∆*_i_* within the first cycle. The stiffness of the specimen decreases rapidly in the initial stage. With the increase in loading displacement amplitude, the stiffness decreases slowly. For the corroded specimens S2, S4, and S7, the stiffness degradation increases slightly with the increase in the average corrosion ratio, but the trend is not obvious. The initial stiffness of the specimens do not change much with the increase in the average corrosion ratio. Due to corrosion damage, the cover concrete failed more easily during the loading process, which reduced the stiffness of the specimen. The stiffness degradation of specimens S3, S4, and S5 gradually increases with the increase in the non-uniform corrosion characteristic value. However, it is not noticeable when the non-uniform corrosion characteristic values are small. The stiffness degradation of S4 is more significant than that of S6, which is mainly due to the larger initial stiffness of the specimen with a larger axial compression ratio.

### 3.5. Energy Dissipation Capacity

**Figure 13:**
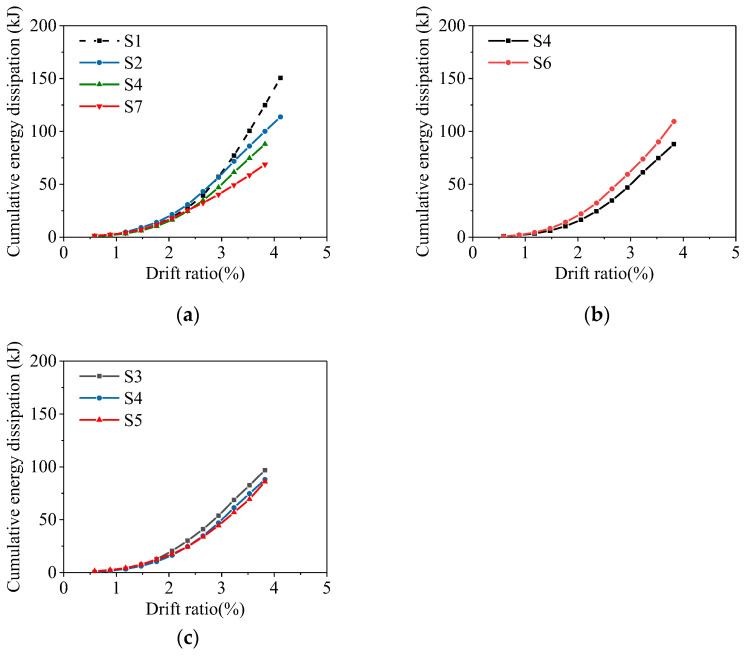
Cumulative energy dissipation of specimens: (**a**) different average corrosion ratio; (**b**) different axial compression ratio; (**c**) different non-uniform corrosion characteristic value.

The cumulative energy dissipation is calculated as the sum of the area enclosed by the hysteresis curve. Figure 13 shows the variation of cumulative energy dissipation with the drift ratio. The cumulative energy dissipation increases with the increase in the drift ratio. For specimens with different average corrosion ratios, S2, S4 and S7, the corrosion ratio has a little effect on the energy dissipation when the drift ratio is small. At a larger drift ratio, the energy dissipation capacity of the specimen significantly decreases with the increase in the average corrosion ratio. Compared to the uncorroded specimen S1, the total cumulative energy dissipation values of S2, S4 and S7 at the ultimate displacement are reduced by 24%, 41%, and 54%, respectively. As the non-uniform corrosion characteristic value increases, the total cumulative energy dissipation values of S3, S4, and S5 at ultimate displacement decrease by 35%, 41%, and 43%, respectively, compared to that of the uncorroded frame, S1. The concentration of damage is detrimental to the energy dissipation of specimens, and the tendency of the effect on energy dissipation becomes insignificant for specimens with large non-uniform corrosion characteristic values. In the small axial compression ratio range, the energy dissipation capacity of the specimen increases with the increase in the axial compression ratio.

### 3.6. Quantitative Evaluation of Corrosion Effect

**Figure 14:**
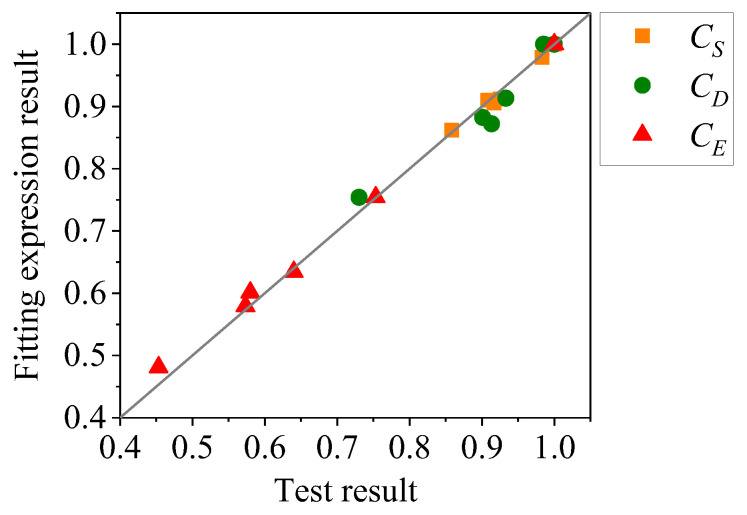
Comparison between fitting expression results and test results.

To quantitatively evaluate the effect of non-uniform corrosion on the seismic performance of RC frames, the following empirical fitting expression for the normalized seismic performance coefficients are derived based on tests results:

(3) $$C_{S}=-3.81\eta^{2}-0.42\rho^{2}+1.60\eta \rho +1$$

(4) $$C_{D}=-1.25\eta^{2}+0.47\rho^{2}-5.17\eta \rho +1$$

(5) $$C_{E}=(-1.37\eta +0.37)e^{-0.2\rho +1.02}$$

where *C_S_* is the coefficient of lateral load bearing capacity which is the bearing capacity of the corroded frame divided by that of uncorroded frame (specimen S1), *C_D_* is the coefficient of displacement ductility, which is the displacement ductility ratio of the corroded frame divided by that of uncorroded frame, and *C_E_* is the coefficient of energy dissipation capacity, which is the cumulative energy dissipation at ultimate displacement of the corroded frame divided by that of uncorroded frame. The comparison between the fitting expression results and the test results is shown in Figure 14. It can be found that the effect of non-uniform corrosion on the energy dissipation capacity of the RC frame is more significant. Due to the limited quantity of samples, the fitting expressions proposed in this study are only applicable to this situation, for which the average corrosion ratio is less than 15% and the non-uniform corrosion characteristics value is less than 0.8.

## 4. Conclusions

Seven non-uniformly corroded RC moment-resisting frame specimens with different parameters were tested under lateral cyclic loading. The following conclusions can be drawn from the test results:

(1) With the increase in the average corrosion ratio, the bearing capacity, energy dissipation capacity, and deformation capacity of the RC frame decrease, and the stiffness degradation becomes more significant. This adverse effect should be considered in the seismic design and assessment of RC structures.
(2) With the increase in the non-uniform corrosion characteristic value, the unidirectional bearing capacity, energy dissipation capacity, and deformation capacity of the RC frame decrease, the stiffness degradation of the RC frame becomes more significant, the damage develops more rapidly, and the damage distribution is more concentrated.
(3) In small axial compression ratio ranges, with the increase in the axial compression ratio, the bearing capacity and energy dissipation capacity of the RC frame increase, and the stiffness degradation is more significant.

Due to the limit of experimental data, the influence of various parameters on the seismic performance of non-uniformly corroded RC frame structures cannot be comprehensively reflected, and a reliable numerical model needs to be established, which can be used to conduct comparative parametric analyses. In addition, the tests in this paper only consider the corrosion of longitudinal reinforcement, and the effect of longitudinal reinforcement and stirrup corrosion on the seismic performance of RC frame structures could be further investigated.

## Figures and Tables

**Table 1:** Main parameters of specimens.

Specimen No.	*η_T_* (%)	*ρ_T_*	*n*	*η_actual_* (%)	*ρ_actual_*
S1	0	-	0.1	-	-
S2	5	0.6	0.1	4.8	0.56
S3	10	0.2	0.1	6.7	0.18
S4	10	0.6	0.1	9.2	0.59
S5	10	0.8	0.1	9.4	0.72
S6	10	0.6	0.2	9.3	0.56
S7	15	0.6	0.1	12.7	0.61

Notes: *η_T_* is the target average corrosion ratio of the longitudinal reinforcement; *ρ_T_* is the target average non-uniform corrosion characteristic value; *n* is the designed axial compressive load ratio; *η_actual_* is the average actual corrosion ratio; *ρ_actual_* is the average actual non-uniform corrosion characteristic value.

**Table 2:** Main test results of specimens.

Specimen No.	Loading Direction	*F_y_* (kN)	∆*_y_* (mm)	*F_m_* (kN)	∆*_m_* (mm)	*F_u_* (kN)	∆*_u_* (mm)	*μ*
S1	Positive	105.3	23.8	119.6	42.9	101.6	73.0	4.04
	Negative	95.6	13.2	121.9	57.1	103.6	76.4	
S2	Positive	92.8	19.2	109.9	36.5	92.5	69.1	3.98
	Negative	64.7	12.4	108.8	47.0	93.4	56.5	
S3	Positive	102.2	22.0	118.4	41.0	100.6	62.0	3.77
	Negative	68.2	13.0	105.5	46.6	89.6	70.1	
S4	Positive	95.2	21.2	110.1	40.5	93.6	57.2	3.69
	Negative	56.9	12.4	102.2	40.6	86.9	65.5	
S5	Positive	86.6	20.4	103.9	30.6	88.5	63.7	3.64
	Negative	61.5	12.1	104.2	30.5	96.8	62.7	
S6	Positive	107.9	21.2	129.1	35.4	109.8	62.1	3.95
	Negative	67.9	11.4	120.6	35.6	102.5	66.8	
S7	Positive	91.8	25.8	109.5	46.0	93.0	62.1	2.95
	Negative	57.7	12.5	93.3	35.1	79.3	51.2	

Notes: *F_y_* and ∆*_y_* are the yielding load and corresponding displacement, respectively; *F_m_* and ∆*_m_* are the peak load and corresponding displacement, respectively; *F_u_* and ∆*_u_* are the ultimate load and corresponding displacement, respectively; *μ* is the displacement ductility ratio.

## Data Availability

Not applicable.

## References

[1] Fu C., Fang D., Ye H., Huang L., Wang J. (2021). Bond Degradation of Non-Uniformly Corroded Steel Rebars in Concrete. Eng. Struct..

[2] Chen E., Berrocal C.G., Fernandez I., Löfgren I., Lundgren K. (2020). Assessment of the Mechanical Behaviour of Reinforcement Bars with Localised Pitting Corrosion by Digital Image Correlation. Eng. Struct..

[3] Chung L., Jay Kim J., Yi S. (2008). Bond Strength Prediction for Reinforced Concrete Members with Highly Corroded Reinforcing Bars. Cem. Concr. Compos..

[4] Ruggieri S., Porco F., Uva G. (2020). A Practical Approach for Estimating the Floor Deformability in Existing Rc Buildings: Evaluation of the Effects in the Structural Response and Seismic Fragility. Bull. Earthq. Eng..

[5] Ruggieri S., Calò M., Cardellicchio A., Uva G. (2022). Analytical-Mechanical Based Framework for Seismic Overall Fragility Analysis of Existing Rc Buildings in Town Compartments. Bull. Earthq. Eng..

[6] Yadav D., Kwatra N., Agarwal P. (2021). Comparative Post-Yield Performance Evaluation of Flexure Member with Corroded Reinforcement. Struct. Infrastruct. Eng..

[7] Huang L., Ye H., Jin X., Jin N., Xu Z. (2020). Corrosion-Induced Shear Performance Degradation of Reinforced Concrete Beams. Constr. Build. Mater..

[8] Firouzi A., Taki A., Mohammadzadeh S. (2019). Time-Dependent Reliability Analysis of RC Beams Shear and Flexural Strengthened with CFRP Subjected to Harsh Environmental Deteriorations. Eng. Struct..

[9] Ma Y., Che Y., Gong J. (2012). Behavior of Corrosion Damaged Circular Reinforced Concrete Columns Under Cyclic Loading. Constr. Build. Mater..

[10] Shang Z., Zheng S., Zheng H., Li Y., Dong J. (2022). Seismic Behavior and Damage Evolution of Corroded RC Columns Designed for Bending Failure in an Artificial Climate. Structures.

[11] Dang V.H., François R. (2014). Prediction of Ductility Factor of Corroded Reinforced Concrete Beams Exposed to Long Term Aging in Chloride Environment. Cem. Concr. Compos..

[12] Okada K., KobayashiI K., Miyagawa T. (1988). Influence of Longitudinal Cracking Due to Reinforcement Corrosion on Characteristics of Reinforced Concrete Members. ACI Struct. J..

[13] Torres-Acosta A.A., Fabela-Gallegos M.J., Muñoz-Noval A., Vázquez-Vega D., Hernandez-Jimenez J.R., Martínez-Madrid M. (2004). Influence of Corrosion on the Structural Stiffness of Reinforced Concrete Beams. Corrosion.

[14] Meda A., Mostosi S., Rinaldi Z., Riva P. (2014). Experimental Evaluation of the Corrosion Influence on the Cyclic Behaviour of RC Columns. Eng. Struct..

[15] Goksu C., Ilki A. (2016). Seismic Behavior of Reinforced Concrete Columns with Corroded Deformed Reinforcing Bars. ACI Struct. J..

[16] Biswas R.K., Iwanami M., Chijiwa N., Uno K. (2020). Effect of Non-Uniform Rebar Corrosion on Structural Performance of RC Structures: A Numerical and Experimental Investigation. Constr. Build. Mater..

[17] Chen J., Zhang W., Gu X. (2019). Modeling Time-Dependent Circumferential Non-Uniform Corrosion of Steel Bars in Concrete Considering Corrosion-Induced Cracking Effects. Eng. Struct..

[18] Su R.K.L., Zhang Y. (2019). A Novel Elastic-Body-Rotation Model for Concrete Cover Spalling Caused by Non-Uniform Corrosion of Reinforcement. Constr. Build. Mater..

[19] Zhao Y., Karimi A.R., Wong H.S., Hu B., Buenfeld N.R., Jin W. (2011). Comparison of Uniform and Non-Uniform Corrosion Induced Damage in Reinforced Concrete Based on a Gaussian Description of the Corrosion Layer. Corros. Sci..

[20] Zhang M., Song H., Lim S., Akiyama M., Frangopol D.M. (2019). Reliability Estimation of Corroded RC Structures Based on Spatial Variability Using Experimental Evidence, Probabilistic Analysis and Finite Element Method. Eng. Struct..

[21] Zhang M., Nishiya N., Akiyama M., Lim S., Masuda K. (2020). Effect of the Correlation of Steel Corrosion in the Transverse Direction Between Tensile Rebars on the Structural Performance of RC Beams. Constr. Build. Mater..

[22] Ye Z., Zhang W., Gu X. (2018). Deterioration of Shear Behavior of Corroded Reinforced Concrete Beams. Eng. Struct..

[23] Yuan W., Guo A., Li H. (2017). Experimental Investigation on the Cyclic Behaviors of Corroded Coastal Bridge Piers with Transfer of Plastic Hinge Due to Non-Uniform Corrosion. Soil Dyn. Earthq. Eng..

[24] Li D., Wei R., Xing F., Sui L., Zhou Y., Wang W. (2018). Influence of Non-Uniform Corrosion of Steel Bars on the Seismic Behavior of Reinforced Concrete Columns. Constr. Build. Mater..

[25] (2016). Code for Seismic Design of Buildings.

[26] Lund University (2001). Contecvet—A Validated Users Manual for Assessing the Residual Service Life of Concrete Structures.

[27] Liu X., Jiang H., He L. (2017). Experimental Investigation on Seismic Performance of Corroded Reinforced Concrete Moment-resisting Frames. Eng. Struct..

[28] Park R. (1989). Evaluation of Ductility of Structures and Structural Assemblages from Laboratory Testing. Bull. N. Z. Soc. Earthq. Eng..

